# The Impact of Different Surgical Modalities for Hysterectomy on Satisfaction and Patient Reported Outcomes

**DOI:** 10.2196/ijmr.3160

**Published:** 2014-07-17

**Authors:** Michael C Pitter, Christopher Simmonds, Usha Seshadri-Kreaden, Helen B Hubert

**Affiliations:** ^1^Newark Beth Israel Medical CenterMinimally Invasive & Gynecologic Robotic SurgeryNewark, NJUnited States; ^2^Intuitive Surgical IncMarketing ServicesSunnyvale, CAUnited States; ^3^Intuitive Surgical IncDepartment of Clinical AffairsSunnyvale, CAUnited States; ^4^Stanford University School of Medicine EmeritaDepartment of MedicineStanford, CAUnited States

**Keywords:** hysterectomy, vaginal hysterectomy, robotics, laparoscopy, patient satisfaction, patient outcomes assessment, Internet

## Abstract

**Background:**

There is an ongoing debate regarding the cost-benefit of different surgical modalities for hysterectomy. Studies have relied primarily on evaluation of clinical outcomes and medical expenses. Thus, a paucity of information on patient-reported outcomes including satisfaction, recovery, and recommendations exists.

**Objective:**

The objective of this study was to identify differences in patient satisfaction and recommendations by approach to a hysterectomy.

**Methods:**

We recruited a large, geographically diverse group of women who were members of an online hysterectomy support community. US women who had undergone a benign hysterectomy formed this retrospective study cohort. Self-reported characteristics and experiences were compared by surgical modality using chi-square tests. Outcomes over time were assessed with the Jonkheere-Terpstra trend test. Logistic regression identified independent predictors of patient satisfaction and recommendations.

**Results:**

There were 6262 women who met the study criteria; 41.74% (2614/6262) underwent an abdominal hysterectomy, 10.64% (666/6262) were vaginal, 27.42% (1717/6262) laparoscopic, 18.94% (1186/6262) robotic, and 1.26% (79/6262) single-incision laparoscopic. Most women were at least college educated (56.37%, 3530/6262), and identified as white, non-Hispanic (83.17%, 5208/6262). Abdominal hysterectomy rates decreased from 68.2% (152/223) to 24.4% (75/307), and minimally invasive surgeries increased from 31.8% (71/223) to 75.6% (232/307) between 2001 or prior years and 2013 (*P*<.001 all trends). Trends in overall patient satisfaction and recommendations showed significant improvement over time (*P*<.001).There were differences across the surgical modalities in all patient-reported experiences (ie, satisfaction, time to walking, driving and working, and whether patients would recommend or use the same technique again; *P*<.001). Significantly better outcomes were evident among women who had vaginal, laparoscopic, and robotic procedures than among those who had an abdominal procedure. However, robotic surgery was the only approach that was an independent predictor of better patient experience; these patients were more satisfied overall (odds ratio [OR] 1.31, 95% CI 1.13-1.51) and on six other satisfaction measures, and more likely to recommend (OR 1.64, 95% CI 1.39-1.94) and choose the same modality again (OR 2.07, 95% CI 1.67-2.57). Abdominal hysterectomy patients were more dissatisfied with outcomes after surgery and less likely to recommend (OR 0.36, 95% CI 0.31-0.40) or choose the same technique again (OR 0.29, 95% CI 0.25-0.33). Quicker return to normal activities and surgery after 2007 also were independently associated with better overall satisfaction, willingness to recommend, and to choose the same surgery again.

**Conclusions:**

Consistent with other US data, laparoscopic and robotic hysterectomy rates increased over time, with a concomitant decline in abdominal hysterectomy. While inherent shortcomings of this retrospective Web-based study exist, findings show that patient experience was better for each of the major minimally invasive approaches than for abdominal hysterectomy. However, robotic-assisted hysterectomy was the only modality that independently predicted greater satisfaction and willingness to recommend and have the same procedure again.

## Introduction

The growth of online support communities and patient engagement on the Internet has provided researchers with unique opportunities to study patient reported outcomes. Population groups can now be readily identified on the Web to study outcomes following disease diagnosis or treatment. Despite their potential limitations, Internet-based studies are able to gather data from a sizeable and geographically diverse patient group at relatively low cost. We used the advantages of this type of data collection to study patient reported experiences following different surgical modalities for hysterectomy.

There has been an ongoing debate regarding the benefits of different surgical techniques for hysterectomy. Many studies attempting to address this question have compared only clinical outcomes, while others have included direct in-hospital costs of the procedures [1-4]. Minimally invasive compared with open approaches for hysterectomy have often been shown to offer somewhat better clinical outcomes, but sometimes at a higher per patient cost [1-6].

More recently, a large study of health insurance claims data linked with workplace absenteeism showed that minimally invasive surgery compared with the open, standard approach for certain conditions resulted in significantly lower health plan spending and significantly fewer days of absence from work over a 1-year peri- and postoperative period [7]. These findings were evident specifically for uterine fibroid resection, the only gynecologic surgery studied. The authors suggest that the policy implications of their results indicate a need for a broader scope of outcomes in the evaluation of technologies, not limited to clinical findings or direct medical expenses.

The present study adds to this ongoing debate by suggesting that patient satisfaction and return to normal activities should also be considered in the equation regarding the cost-benefit of different surgical modalities. Our study describes the use of abdominal, vaginal, laparoscopic, robotic-assisted, and single-incision laparoscopic techniques for benign hysterectomy over time in a large online patient community seeking and providing information about hysterectomy and related aspects of women’s health. Since benign hysterectomy has seen a significant change in treatment choice over the past decade, this study importantly compares time trends in the use of the different modalities as well as differences in self-reported patient recovery, satisfaction, and recommendations. The independent associations of surgical approach and recovery with overall patient satisfaction and recommendations are further explored.

## Methods

### Participants

Participants in this study were identified through HysterSisters.com, a large online community of women who give and receive support and resources for hysterectomy decisions, treatments, and recovery. HysterSisters.com has been certified by the Health on the Net Foundation to “promote and guide the deployment of useful and reliable online health information, and its appropriate and efficient use.” The website was launched in 1998 and has registered close to 300,000 women since that time. The HysterSisters’ privacy policy indicates that research related to women’s health is sometimes conducted on the site “…to identify unmet needs of women through aggregate reporting of women's experiences, opinions and therapeutic treatments.” HysterSisters’ founder agreed to the plans and purpose of this study.

In early February 2013, all members who were still registered to receive the HysterSisters’ Web-based newsletter and had valid email addresses were invited to participate in this study. All contact with the membership was done through HysterSisters so that no one on the study team had access to individual email addresses. Only women in the United States who linked to and completed the Web-based survey and indicated that they already had a hysterectomy for benign indications are included in this report.

### Data Collection

Data were collected with the assistance of HysterSisters.com and MarketTools software over the period February 4^th^ to February 13^th^ 2013. A link on the February HysterSisters’ newsletter invited recipients to participate in the hysterectomy survey. A specific email went out from the HysterSisters’ founder 2 days later inviting women who had a hysterectomy or were thinking about having one to provide their input in a short 5 to 7 minute survey. A follow-up email, identical to the previous one, was sent on February 11th. Women accessed the study questionnaire through a link on one of the communications. Cookies were used to track survey access so that all completed questionnaires were from unique respondents. This study received an exemption from the requirement of institutional review board (IRB) approval from Quorum Review IRB and this exemption is on file.

The data collection tool was developed to adequately address questions in the study protocol. Based on past experience, the Web-based survey was tested via the Internet to ensure proper function including the adaptive questions that created a skip pattern depending on an individual’s responses. Each participant was shown four to five screens and answered up to 24 questions with each screen containing two to six items. Respondents were able to go back to review and change answers.

All questions on the survey had categorical multiple-choice responses. They included whether the participant ever had surgery to remove the uterus (hysterectomy), the primary reason (benign/noncancerous or cancerous conditions with examples), the type of hysterectomy with explanations (abdominal, vaginal, laparoscopically-assisted vaginal hysterectomy, laparoscopic hysterectomy, robotic-assisted hysterectomy, single-incision laparoscopic hysterectomy, and not sure), and the year, beginning with 2001 or earlier up to 2013. Respondents also indicated if they had had specific abdominopelvic or gynecologic surgeries prior to their hysterectomy.

On a 5-point (Likert type) scale, participants rated how satisfied (extremely satisfied, very satisfied, somewhat satisfied, not satisfied, extremely dissatisfied) they were overall with the surgery and with specific aspects of hysterectomy (invasiveness, complications, length of hospital stay, pain and discomfort, recurrence of problem, and time until return to normal activities) and how likely they would be to recommend the same type of hysterectomy to someone else. In addition, women were asked what modality they would choose if they had to do the surgery all over again. Respondents also reported the amount of time it took them to return to normal activities including walking, driving, and getting back to work.

Self-reported sociodemographic information was collected on age group, education, family income, race/ethnicity, urban/suburban/rural residence, and type of health insurance (private, Medicare, Medicaid, other). No personal identifiers were obtained. These data were stored in a centralized database with password-protected access for study researchers only.

### Statistical Analysis

All analyses excluded the small number of women who were unsure of the surgical technique. Study categories included abdominal, vaginal, laparoscopic (combining laparoscopic and laparoscopically-assisted vaginal hysterectomy), robotic-assisted, and single-incision laparoscopic hysterectomy. For most analyses, year of hysterectomy was grouped into three periods (2007 or prior, 2008-2010, 2011-2013). It was important that all types of surgeries were performed in each time period to reduce possible confounding of time of surgery with approach. Thus, the first period was defined to include the newest robotic approach, first reported by this cohort in 2006. The years 2008 to 2013 were split evenly into two 3-year periods.

Chi-square tests were used to compare outcomes over the different surgical modalities. Trends over time were assessed with the Jonkheere-Terpstra test for trend. Logistic regression was used to describe the associations of surgical technique with patient experience, adjusted for age and time period. Multivariable, forward, stepwise logistic regression was employed to identify characteristics that were independently associated with important outcomes after surgery: (1) how satisfied the respondents were with the overall and specific results after surgery (time to return to normal activities, pain and discomfort, invasiveness, complications, length of hospital stay, and recurrence), (2) how likely they would be to recommend their surgical approach to another person, and (3) whether they would choose the same technique again. The variables considered for entry into the logistic model were chosen a priori without considering results of univariate or bivariate analyses. These included patient age group (<40, 40-49, 50-59, 60+), education (high school or less, some college, college graduate, graduate school or higher), family income (<$50,000, $50,000-$75,000, $76,000-$125,000, >$125,000), race/ethnicity (white, non-Hispanic, African American, Hispanic, other), community type (urban, suburban, rural), type of hysterectomy, time periods of surgery (≤2007, 2008-2010, 2011-2013), and prior abdominopelvic surgery (yes/no). Recovery time regarding walking (within 1-2 days, yes/no), driving (within 1 week, yes/no), and returning to work (within 4 weeks, yes/no) was also allowed to enter for some outcomes. No imputed values were used in the regression analyses. Results are presented using the odds ratio (OR) and its 95% confidence limits (95% CI).

All analyses were performed using SAS version 9.2. Given the large data set analyzed, a two-sided *P*<.01 was used to determine statistical significance.

## Results

### Online Recruitment

The HysterSisters’ February newsletter was sent to 134,618 members of the online community. Two dedicated study invitation emails subsequently went out to those who had valid email addresses (114,116/134,618, 84.77%), as determined by the newsletter mailing. At least one of the mailings was opened by 55.29% (63,095/114,116) of women. Of those, 18.53% (11,694/63,095) clicked on the survey link and 17.80% (11,232/63,095) began to complete the questionnaire. There were 121 women screened out on the first question by responding that they never had and were not planning to have a hysterectomy and 1934 completed only part of the survey. The questionnaire was completed in full by 78.48% (9177/11,694) of those who clicked on the link or 14.54% (9177/63,095) of those who opened one of the emails. This paper describes the results of analysis of all US respondents who had undergone a benign hysterectomy and specified the surgical modality (6262). The techniques included 41.74% (2614/6262) abdominal, 10.64% (666/6262) vaginal, 27.42% (1717/6262) laparoscopic, 18.94% (1186/6262) robotic, and 1.26% (79/6262) single-incision laparoscopic hysterectomies.

### Patient Sociodemographic Characteristics

A majority of women, 77.34% (4843/6262), had their hysterectomies between the ages of 40 and 59 (Table 1). A college or graduate degree was obtained by 56.37% (3530/6262) and yearly family income was below $50,000 in 22.34% (1399/6262). A little more than one-half of the participants, 52.59% (3293/6262), indicated that their family income was over $75,000. The group was predominantly white non-Hispanic, as reported by 83.17% (5208/6262); 8.80% (551/6262) considered themselves African American, and 3.80% (238/6262) Hispanic. Most of the respondents, 53.75% (3366/6262), lived in suburban areas; 18.48% (1157/6262) came from urban cities and 27.77% (1739/6262) were rural dwellers. Health insurance was private, either through an employer or through self-insurance, in 85.47% (5352/6262) of the total group.

**Table 1:** Patient sociodemographic characteristics by surgical approach.

Characteristics Total N=6262		Abdominal N=2614	Vaginal N=666	Laparoscopic N=1717	Robotic N=1186	Single-incision laparoscopic N=79	*P* value^a^
		n (%)	n (%)	n (%)	n (%)	n (%)	
**Age, years**							<.001
	<40	333 (12.74)	110 (16.52)	362 (21.08)	224 (18.89)	10 (12.66)	
	40-49	1097 (41.97)	269 (40.39)	780 (45.43)	599 (50.51)	31 (39.24)	
	50-59	1017 (38.91)	201 (30.18)	502 (29.24)	315 (26.56)	32 (40.51)	
	≥60	167 (6.39)	86 (12.91)	73 (4.25)	48 (4.05)	6 (7.59)	
**Education**							.009
	Middle or high school	362 (13.85)	94 (14.11)	231 (13.45)	119 (10.03)	10 (12.66)	
	Some college	788 (30.15)	223 (33.48)	543 (31.62)	343 (28.92)	19 (24.05)	
	College	954 (36.50)	237 (35.59)	642 (37.39)	478 (40.30)	34 (43.04)	
	Graduate school or higher	510 (19.51)	112 (16.82)	301 (17.53)	246 (20.74)	16 (20.25)	
**Family income**							<.001
	<$50,000	644 (24.64)	139 (20.87)	369 (21.49)	223 (18.80)	24 (30.38)	
	$50,000-$75,000	649 (24.83)	174 (26.13)	447 (26.03)	283 (23.86)	17 (21.52)	
	$76,000-$125,000	817 (31.25)	237 (35.59)	589 (34.30)	427 (36.00)	17 (21.52)	
	>$125,000	504 (19.28)	116 (17.42)	312 (18.17)	253 (21.33)	21 (26.58)	
**Race**							<.001
	White	2099 (80.30)	592 (88.89)	1462 (85.15)	991 (83.56)	64 (81.01)	
	African American	298 (11.40)	30 (4.50)	120 (6.99)	98 (8.26)	5 (6.33)	
	Hispanic	96 (3.67)	16 (2.40)	67 (3.90)	55 (4.64)	4 (5.06)	
	Other	121 (4.63)	28 (4.20)	68 (3.96)	42 (3.54)	6 (7.59)	
**Community type**							.04
	Urban	500 (19.13)	111 (16.67)	300 (17.47)	230 (19.39)	16 (20.25)	
	Suburban	1408 (53.86)	341 (51.20)	912 (53.12)	664 (55.99)	41 (51.90)	
	Rural	706 (27.01)	214 (32.13)	505 (29.41)	292 (24.62)	22 (27.85)	
**Insurance type**							.10
	Private	2217 (84.81)	551 (82.73)	1470 (85.61)	1045 (88.11)	68 (86.08)	
	Medicare	81 (3.10)	33 (4.95)	54 (3.15)	29 (2.45)	3 (3.80)	
	Medicaid	60 (2.30)	19 (2.85)	35 (2.04)	16 (1.35)	1 (1.27)	
	Other	256 (9.79)	63 (9.46)	158 (9.20)	96 (8.09)	7 (8.86)	

^a^ *P* value based on the overall chi-square test of the characteristic by surgical modality.

Comparison of the different treatment approaches on sociodemographic characteristics showed that patients differed significantly (*P*<.01) across techniques on age group, education, family income, and race/ethnicity (Table 1). Patients who had undergone laparoscopic or robotic hysterectomy were similar in age (*P*=.062), but younger than women who had abdominal or vaginal surgeries (*P*<.001). The group who had robotic surgery was also younger than those who had the single-incision laparoscopic approach (*P*=.01). A higher percentage of patients who had single-site laparoscopies were greater than 50 years of age when compared with the other surgical groups.

The most consistent finding with regard to socioeconomic status was that patients who had a robotic hysterectomy were better educated (*P*=.003) and had higher family income (*P*<.001) than women who had the abdominal approach (Table 1). The lowest percentage of white women was found in the group who had abdominal surgery (80.30%, 2099/2614; *P*<.01 for comparisons with each of the other modalities except single-incision laparoscopy where there was no difference).

### Time Trends in Modalities for Hysterectomy

**Figure 1:**
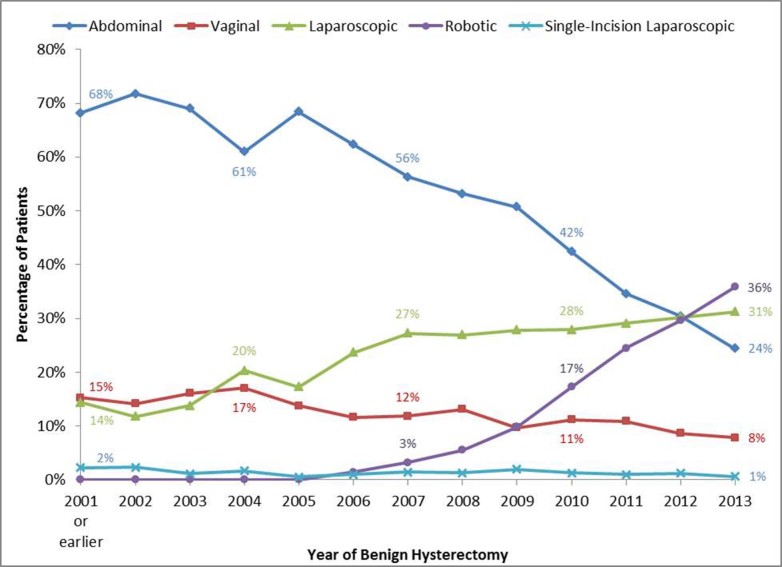
Trends in the use of each surgical approach over time.

Trends over time indicated that the use of abdominal and vaginal approaches significantly declined between 2001 or earlier years and 2013 (*P*<.001 for both trends, Figure 1). Abdominal hysterectomy rates fell from 68.2% (152/223) to 24.4% (75/307) and vaginal rates from 15.2% (34/223) to 7.8% (24/307) over time. The use of laparoscopic surgery significantly increased from 14.3% (32/223) to 31.3% (96/307) and robotic surgery from 0% (0/223) (prior to its approval and introduction in 2005) to 35.8% (110/307) in 2013 (*P*<.001 for both trends). Single-incision laparoscopic hysterectomy rates were very low and basically unchanged over time (5/223, 2.2% in 2001 or prior years to 2/307, 0.7% in 2013). Thus, in this study, minimally invasive hysterectomy increased from 31.8% (71/223) of procedures in 2001 or earlier to 75.6% (232/307) of surgeries in 2013.

### Patient Reported Experience: Satisfaction, Recovery, and Recommendations After Hysterectomy

There were significant differences in each measured outcome across the surgical modalities as described in Table 2 (*P*<.001). Logistic regression analysis, including three covariates (age group, time period, and approach to hysterectomy), was used to describe associations of procedure and time period with each outcome. Findings indicated that there was less overall satisfaction and more dissatisfaction in the abdominal hysterectomy group when compared with the vaginal (*P*=.009), laparoscopic (*P*<.001), and robotic (*P*<.001) surgical groups. Extreme satisfaction rates ranged from 41.35% (1081/2614) in the abdominal to 55.73% (661/1186) in the robotic hysterectomy group. These analyses also showed that overall satisfaction significantly increased between time periods 1 and 3 (*P*<.001).

Less satisfaction and more dissatisfaction with time needed to return to normal activities was also evident among women who had an abdominal hysterectomy when compared with those who had vaginal, laparoscopic, and robotic minimally invasive surgery (all *P*<.001). Rates of extreme satisfaction varied from 18.63% (487/2614) among those who had abdominal procedures to 39% (31/79) in the single-incision laparoscopic group.

Logistic regression results regarding satisfaction with pain and discomfort after hysterectomy again indicated that women who had the abdominal approach were less satisfied when compared with those in each of the other groups, except single-incision (all *P*<.001). These analyses also showed that satisfaction was significantly less in time period 1 compared with period 3 and greater at older ages compared with younger (both *P*<.001).

Similarly, significantly less satisfaction with invasiveness of the surgery, complications associated with the hysterectomy, and length of hospital stay were seen with abdominal hysterectomy compared to vaginal, laparoscopic, and robotic approaches (all *P*<.001). More dissatisfaction was evident in time period 1 than in period 3 (all *P*<.002).

Satisfaction with recurrence of the problem after hysterectomy was greater for those who had robotic surgery only (*P*<.001), when comparing the abdominal approach to the other minimally invasive procedures. Less satisfaction was seen in the earliest time period and at younger than older ages.

The abdominal hysterectomy group also was less likely to return to walking within 2 days, to driving within 1 week and to working within 4 weeks than the vaginal (*P*=.008), laparoscopic (*P*<.001), and robotic groups (*P*<.001). In addition, those who were younger (*P*=.001) and had surgery most recently (*P*=.006) returned to walking sooner. Rates of early return to driving and work were highest for those who had the single-incision laparoscopic hysterectomy, 39.7% (31/78) and 57.8% (37/64), respectively.

Recommendation of the surgical approach to another and choosing the same technique again followed similar trends by modality (Table 2). Patients who had an abdominal hysterectomy were significantly less likely to recommend that approach or choose it again than patients who had one of the minimally invasive procedures (all *P*<.001). Those who had surgery in the earliest time period versus the most recent were less likely to recommend their approach (*P*<.001). Women who had surgery in the most recent time period versus time periods 1 or 2 were significantly more likely to choose their procedure again (both *P*<.001).

Rates of definitely recommending the same procedure ranged from 27.74% (725/2614) in the abdominal to 69.81% (828/1186) in the robotic hysterectomy group and rates for choosing the same hysterectomy procedure again varied from 39.02% (1020/2614) in the abdominal to 86.85% (1030/1186) in the robotic surgery group.

**Table 2:** Patient satisfaction, recovery, and recommendations by surgical technique.

Characteristics Total N=6262		Abdominal N=2614	Vaginal N=666	Laparoscopic N=1717	Robotic N=1186	Single-incision laparoscopic N=79	*P* value^a^
		n (%)	n (%)	n (%)	n (%)	n (%)	
**Overall hysterectomy results**							<.001
	Extremely satisfied	1081 (41.35)	317 (47.60)	862 (50.20)	661 (55.73)	40 (50.63)	
	Satisfied	1346 (51.49)	305 (45.80)	762 (44.38)	483 (40.73)	31 (39.24)	
	Dissatisfied	187 (7.15)	44 (6.61)	93 (5.42)	42 (3.54)	8 (10.13)	
**Time to return to normal activities**							<.001
	Extremely satisfied	487 (18.63)	185 (27.78)	497 (28.95)	400 (33.73)	31 (39.24)	
	Satisfied	1640 (62.74)	395(59.31)	989 (57.60)	635 (53.54)	40 (50.63)	
	Dissatisfied	487 (18.63)	86 (12.91)	231 (13.45)	151 (12.73)	8 (10.13)	
**Pain and discomfort after hysterectomy**							<.001
	Extremely satisfied	445 (17.02)	189 (28.38)	444 (25.86)	413 (34.82)	24 (30.38)	
	Satisfied	1539 (58.88)	355 (53.30)	1015 (59.11)	616 (51.94)	45 (56.96)	
	Dissatisfied	630 (24.10)	122 (18.32)	258 (15.03)	157 (13.24)	10 (12.66)	
**Invasiveness of hysterectomy**							<.001
	Extremely satisfied	475 (18.17)	291 (43.69)	735 (42.81)	616 (51.94)	41 (51.90)	
	Satisfied	1782 (68.17)	339 (50.90)	927 (53.99)	536 (45.19)	35 (44.30)	
	Dissatisfied	357 (13.66)	36 (5.41)	55 (3.20)	34 (2.87)	3 (3.80)	
**Complications associated with hysterectomy**							<.001
	Extremely satisfied	777 (29.72)	240 (36.04)	645 (37.57)	527 (44.44)	40 (50.63)	
	Satisfied	1342 (51.34)	316 (47.45)	849 (49.45)	527 (44.44)	31 (39.24)	
	Dissatisfied	495 (18.94)	110 (16.52)	223 (12.99)	132 (11.13)	8 (10.13)	
**Length of hospital stay**							<.001
	Extremely satisfied	656 (25.10)	265 (39.79)	735 (42.81)	602 (50.76)	39 (49.37)	
	Satisfied	1716 (65.65)	347 (52.10)	870 (50.67)	530 (44.69)	37 (46.84)	
	Dissatisfied	242 (9.26)	54 (8.11)	112 (6.52)	54 (4.55)	3 (3.80)	
**Recurrence of problem after hysterectomy**							<.001
	Extremely satisfied	1384 (52.95)	364 (54.65)	976 (56.84)	722 (60.88)	43 (54.43)	
	Satisfied	1058 (40.47)	240 (36.04)	601 (35.00)	406 (34.23)	27 (34.18)	
	Dissatisfied	172 (6.58)	62 (9.31)	140 (8.15)	58 (4.89)	9 (11.39)	
**Return to walking**							<.001
	Within 2 days	1036 (39.74)	302 (45.62)	870 (50.97)	645 (54.94)	34 (43.04)	
	After 2 days	1571 (60.26)	360 (54.38)	837 (49.03)	529 (45.06)	45 (56.96)	
	N/A	7	4	10	12	0	
**Return to driving**							<.001
	Within 1 week	307 (12.23)	196 (30.43)	561 (33.71)	398 (34.43)	31 (39.74)	
	After 1 week	2204 (87.77)	448 (69.57)	1103 (66.29)	758 (65.57)	47 (60.26)	
	N/A	103	22	53	30	1	
**Return to work**							<.001
	Within 4 weeks	458 (20.70)	211 (40.81)	722 (50.10)	534 (53.67)	37 (57.81)	
	After 4 weeks	1755 (79.30)	306 (59.19)	719 (49.90)	461 (46.33)	27 (42.19)	
	N/A	401	149	276	191	15	
**Would recommend the same type of hysterectomy**							<.001
	Definitely	725 (27.74)	376 (56.46)	984 (57.31)	828 (69.81)	48 (60.76)	
	Likely	1241 (47.48)	223 (33.48)	613 (35.70)	302 (25.46)	22 (27.85)	
	Unlikely	648 (24.79)	67 (10.06)	120 (6.99)	56 (4.72)	9 (11.39)	
**Would choose the same type of hysterectomy again**							<.001
	Yes	1020 (39.02)	482 (72.37)	1231 (71.7)	1030 (86.85)	55 (69.62)	
	No/not sure	1594 (60.98)	184 (27.63)	486 (28.3)	156 (13.15)	24 (30.38)	

^a^ *P* value based on the overall chi-square test of the characteristic by surgical modality.

### Independent Predictors of Patient Satisfaction and Recommendations After Hysterectomy

**Figure 2:**
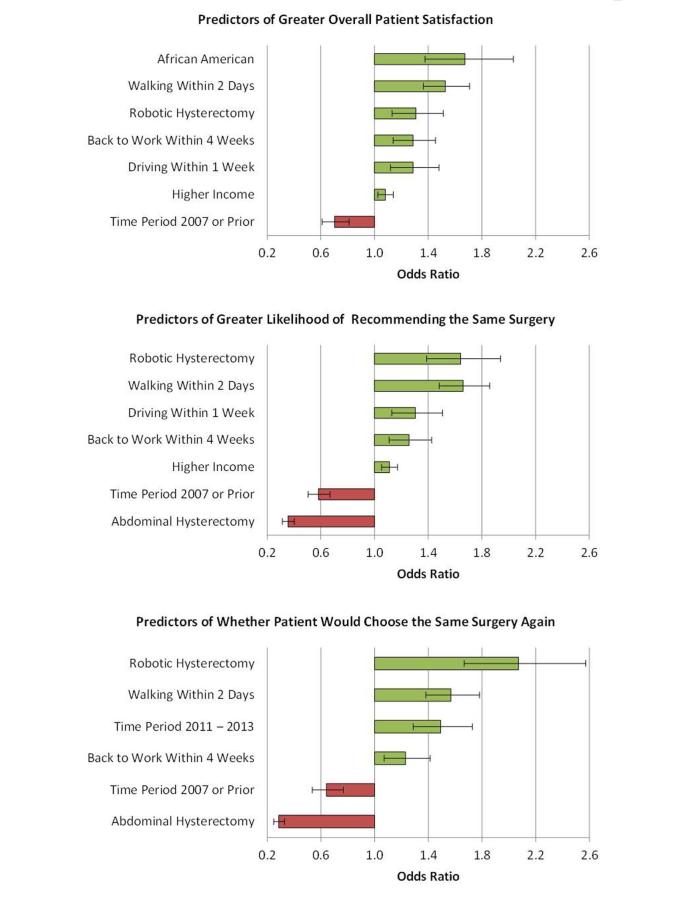
Independent predictors of overall patient satisfaction and recommendations after hysterectomy.

**Figure 3:**
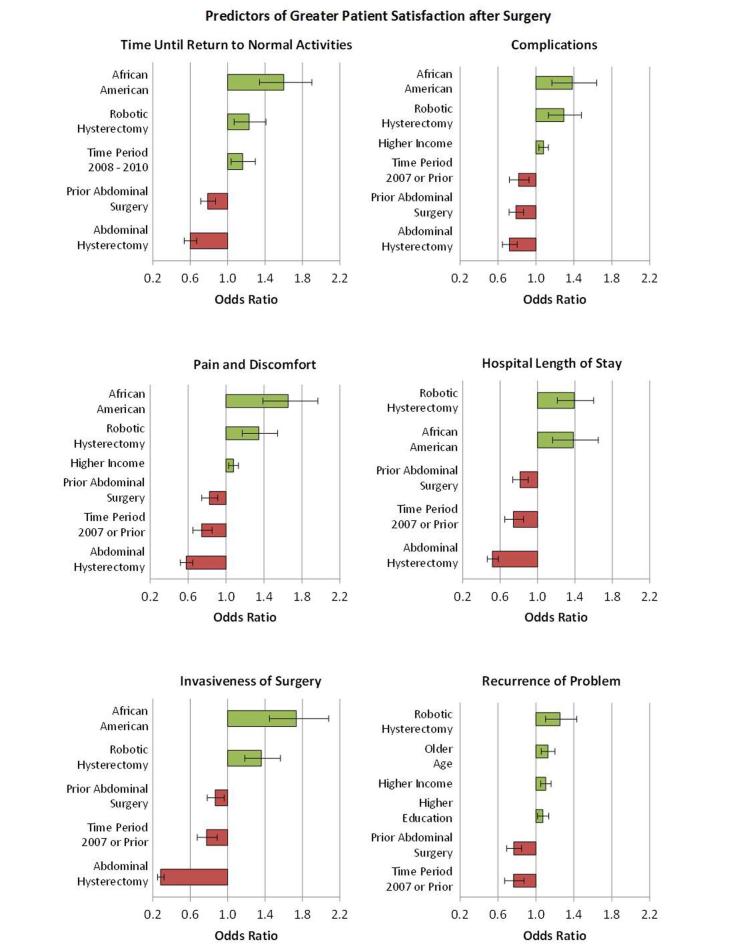
Independent predictors of greater patient satisfaction after hysterectomy.

The multiple impacts of sociodemographic factors, time period of surgery, prior abdominopelvic surgery, patient-reported recovery time, and surgical modality on patient satisfaction and recommendations after hysterectomy were examined in forward, stepwise, multivariable logistic regression as described in the methods. Figure 2 presents the significant independent findings (*P*<.01) for these outcomes: (1) satisfaction with results overall (extremely satisfied, satisfied, dissatisfied), (2) would recommend the procedure to another (definitely, likely, unlikely), (3) would choose the same procedure for oneself again (yes, no). Figure 3 shows findings for satisfaction with: time until return to normal activities, pain and discomfort, invasiveness, complications, length of hospital stay, and recurrence. Single-incision laparoscopic hysterectomy was not included in these analyses due to the small number of surgeries employing this technique.

Independent predictors of being more satisfied overall were African American race (OR 1.67, 95% CI 1.38-2.04; *P*<.001), walking within 2 days after surgery (OR, 1.53, 95% CI 1.36-1.71; *P*<.001), having had a robotic-assisted hysterectomy (OR 1.31, 95% CI 1.13-1.51; *P*<.001), returning to work within 4 weeks after surgery (OR 1.29, 95% CI 1.14-1.46; *P*<.001), driving within 1 week after surgery (OR 1.29, 95% CI 1.12-1.48; *P*<.001) and higher income (OR 1.08 per income level, 95% CI 1.02-1.14; *P*=.005). Having had a hysterectomy in the earliest time period (ie, 2007 or prior) was a predictor of being less satisfied with the overall results of hysterectomy (OR 0.70, 95% CI 0.61-0.81; *P*<.001). Thus, after controlling for the effects of the other significant contributors to overall satisfaction, robotic-assisted hysterectomy was associated with a 31% greater odds of being: (1) extremely satisfied compared with less satisfied or dissatisfied (combined), and (2) extremely satisfied or satisfied (combined) compared with dissatisfied (Figure 2).

Characteristics that were independently and positively associated with a greater likelihood of recommending the same surgery to someone else included having had a robotic-assisted hysterectomy (OR 1.64, 95% CI 1.39-1.94; *P*<.001), walking within 2 days (OR 1.66, 95% CI 1.48-1.86; *P*<.001), driving within 1 week (OR 1.30, 95% CI 1.13-1.51; *P*<.001), returning to work within 4 weeks (OR 1.26; 95% CI 1.11-1.43; *P*<.001), and higher income (OR 1.11, 95% CI 1.05-1.17; *P*<.001). Having undergone an abdominal hysterectomy (OR 0.36, 95% CI 0.31-0.40; *P*<.001) and having had surgery in the earliest time period (OR 0.58, 95% CI 0.51-0.67; *P*<.001) were associated with being less likely to recommend the same surgery to someone else. Robotic-assisted hysterectomy was independently associated with a 64% greater odds and abdominal hysterectomy was associated with a 36% lower odds of definitely recommending the same procedure versus being likely or unlikely (combined) to recommend it, and definitely or likely (combined) versus unlikely to recommend it.

Predictors of patients choosing the same surgery again were having a robotic-assisted hysterectomy (OR 2.07, 95% CI 1.67-2.57; *P*<.001), walking within 2 days (OR 1.57, 95% CI 1.38-1.78; *P*<.001), having undergone surgery in the most recent time period, 2011-2013 (OR 1.49, 95% CI 1.29-1.73; *P*<.001), and returning to work within 4 weeks (OR 1.23, 95% CI 1.07-1.41; *P*=.003). Having undergone an abdominal hysterectomy (OR 0.29, 95% CI 0.25-0.33; *P*<.001) and having had surgery in the earliest time period (OR 0.64, 95% CI 0.54-0.77; *P*<.001) were associated with not choosing the same surgery for oneself again. The odds of abdominal hysterectomy patients choosing the same approach again were 29% of the odds associated with all other modalities. Robotic hysterectomy patients were more than twice as likely as others to choose the same approach again. There were no significant differences in these outcomes by age group, education, urban/rural status, and prior abdominopelvic surgery.

Results of multivariable stepwise logistic regression on components of patient satisfaction are shown in Figure 3. Greater satisfaction with time until return to normal activities was evident among African American women (OR 1.60, 95% CI 1.34-1.90; *P*<.001), those who had a robotic-assisted hysterectomy (OR 1.23, 95% CI 1.07-1.41; *P*=.007), and whose surgery was in the time period 2008-2010 (OR 1.16, 95% CI 1.04-1.30; *P*=.009). Abdominal hysterectomy (OR 0.60, 95% CI 0.54-0.67; *P*<.001) and prior abdominopelvic surgery (OR 0.79, 95% CI 0.71-0.87; *P*<.001) were associated with less satisfaction and, thus, more dissatisfaction.

Being African American (OR 1.65, 95% CI 1.39-1.97; *P*<.001), robotic surgery (OR 1.35, 95% CI 1.17-1.54; *P*<.001), older age (OR 1.14, 95% CI 1.07-1.21; *P*<.001), and higher income (OR 1.08, 95% CI 1.03-1.13; *P*<.001) independently predicted greater satisfaction with pain and discomfort after surgery. Abdominal hysterectomy (OR 0.58, 95% CI 0.52-0.65; *P*<.001), surgery in 2007 or earlier years (OR 0.75, 95% CI 0.65-0.85; *P*<.001), and prior abdominopelvic surgery (OR 0.83, 95% CI 0.75-0.91; *P*<.001) were associated with less satisfaction.

Greater satisfaction with invasiveness of surgery was also associated with being African American (OR 1.73, 95% CI 1.45-2.08; *P*<.001) and having had robotic surgery (OR 1.36, 95% CI 1.18-1.56; *P*<.001). Less satisfaction was evident among those who had an abdominal hysterectomy (OR 0.28, 95% CI 0.25-0.32; *P*<.001), underwent surgery in the earliest time period (OR 0.77, 95% CI 0.68-0.89; *P*<.001), and experienced a prior abdominopelvic procedure (OR 0.86, 95% CI 0.78-0.96; *P*=.007).

Satisfaction with complications was significantly greater among African Americans (OR 1.38, 95% CI 1.17-1.64; *P*<.001) and those who had robotic surgery (OR 1.29, 95% CI 1.13-1.48; *P*<.001) and higher income (OR 1.08, 95% CI 1.03-1.13; *P*=.001). Women who had an abdominal hysterectomy (OR 0.72, 95% CI 0.65-0.80; *P*<.001), prior abdominopelvic surgery (OR 0.79, 95% CI 0.72-0.87; *P*<.001), and surgery in the earliest time period (OR 0.82, 95% CI 0.72-0.93; *P*=.002) were significantly less satisfied.

Robotic surgery (OR 1.39, 95% CI 1.21-1.60; *P*<.001) and being African American (OR 1.38, 95% CI 1.16-1.65; *P*<.001) predicted greater satisfaction with length of hospital stay. Abdominal hysterectomy (OR 0.52, 95% CI 0.46-0.58; *P*<.001), the earliest time period of surgery (OR 0.74, 95% CI 0.65-0.85; *P*<.001), and prior abdominopelvic surgery (OR 0.81, 95% CI 0.74-0.90; *P*<.001) predicted greater dissatisfaction.

Women who had robotic surgery (OR 1.26, 95% CI 1.10-1.43; *P*<.001), who were older in age (OR 1.13, 95% CI 1.06-1.20; *P*<.001), and had higher income (OR 1.10, 95% CI 1.05-1.16; *P*<.001), and education (OR 1.07, 95% CI 1.02-1.14; *P*=.009) were significantly more satisfied with recurrence of their problem after surgery. Those who had surgeries in the earliest time period (OR 0.77, 95% CI 0.67-0.88; *P*=.002) and prior abdominopelvic surgery (OR 0.77, 95% CI 0.69-0.85; *P*<.001) were less satisfied.

## Discussion

### Principal Results

During the study period, the rates of abdominal and vaginal hysterectomy declined with a concomitant increase in laparoscopic and robotic hysterectomy. There was no change noted in the relative number of single-site laparoscopic hysterectomy procedures being performed. In fact, there was pivotal point in the year 2012 where usage of the three major modalities intersected. In the following and final year of the study, it appears that robotic-assisted laparoscopic hysterectomy was reported by more women (35.8%, 110/307) than open abdominal hysterectomy (24.4%, 75/307) and slightly more than laparoscopic hysterectomy (31.3%, 96/307). Improvements in all patient experiences over time as measured by satisfaction with results, timely return to work, quicker return to normal activities such as walking and driving may be partially attributed to the increasing rates of minimally invasive surgery.

Robotic-assisted laparoscopic hysterectomy is the only modality that was an independent predictor of better patient experience with better overall satisfaction, better satisfaction with specific outcomes, and greater willingness to recommend and choose the same technique again even after accounting for the effects of other important predictors. Abdominal hysterectomy was associated with a worse patient experience relative to the other types of procedures.

Analyses suggest that possible reasons for significantly better overall satisfaction with robotic than other hysterectomy procedures, despite similar invasiveness of the robotic and laparoscopic procedures and less invasiveness of the vaginal approach, include greater satisfaction with return to normal activities, pain and discomfort, perceived invasiveness, complications, length of hospital stay, and recurrence of problems among women who had the robotic approach.

Early return to activities including walking, driving, and work were also independent predictors of overall satisfaction and the likelihood of recommending the same procedure. African Americans reported greater overall satisfaction after hysterectomy due to greater satisfaction with time until return to normal activities, with pain and discomfort, perceived invasiveness of surgery, complications, and length of hospital stay. However, these findings did not translate into greater likelihood of their recommending the same approach or choosing it again.

### Limitations

As a retrospective study, the potential for unmeasured biases always exists. In the present study, some of these were balanced by the fact that the data were obtained from women who were part of a database and had surgery at many different hospitals, performed by many different surgeons across the United States. Thus, the limitations and biases from unique surgical programs were eliminated. Difficulties in recall may have played a role in answering certain survey questions, particularly about surgeries from the earliest years. However, it is unknown if specific recall biases occurred that would have affected study results. Participants were given the opportunity to indicate “not sure” on any questions asking about type of procedure. Furthermore, patients who had procedures in the earlier years reported less satisfaction than those who had procedures in later periods, even in multivariable analysis. One might expect that recall of such issues in the earlier years would be diminished and make it more difficult to find such significant differences.

We did not collect data on the primary reason for the hysterectomy, severity of disease, whether ovaries or the cervix were removed, number of complications from surgery, patient characteristics like obesity or uterine size, comorbidity, functional status, contraindications for specific modalities, and employment or self-employment, any of which may been “unobserved” influences on choice of technique or patient experience over time. These aspects could not be considered or adjusted for in multivariable analysis. We had no way of ascertaining if, for example, women who had robotic surgery were more actively involved in the choice of the approach and, therefore, were more satisfied with the outcome. While differences observed in sociodemographic groups by modality could influence results of patient experience, these were controlled for as well as possible in multivariable logistic regression analysis using income and education as surrogates.

In terms of recruitment for an Web-based survey, this study had a good response rate. Among those who clicked on the link to the survey, 78.48% (9177/11,694) continued on to completion. We also recruited a large study population, a total of 9177, in a short time frame of 10 days. In a randomized study of the reliability of Internet versus mailed questionnaires for assessing health, activity level, disability, and health care utilization, Ritter et al [8] found that participation rates were at least as good, if not better, for those assigned to the Internet compared with those assigned to the mailed questionnaires with less recruitment effort.

However, these results may not be generalizable to the population of all hysterectomy patients since, for example, data suggest that Internet users may be younger, better educated, and have higher family income than the general population [9]. Nevertheless, any such selection bias would likely be the same across the surgical modalities so as not to impact results of comparisons by approach. In addition, influences of age and socioeconomic status were considered, and adjusted for when appropriate, in multivariable analysis of all outcomes. The positive aspects of using an online community for our research, ready availability, large size, geographic diversity, and low cost, could outweigh the aforementioned limitations.

This study survey was not measuring clinical outcomes that are sometimes inaccurately self-reported by patients. Participants did need to reliably report the type of hysterectomy they had undergone, but, as mentioned, were also given the option of choosing “not sure.” We had no means to validate this self-reported information. Research suggests that patients can reliably self-report many, especially chronic, medical conditions. For example, Cascade et al [10] compared patient reports of gout with medical record diagnosis and found a 97.4% confirmation rate. Studies of treatments for breast cancer in women have shown high reliability. In particular, Gupta et al [11] found a 94% concordance rate between self-report and medical record for type of surgery conducted for breast cancer. Maunsell et al [12] showed very high agreement between self-report at 3-years post diagnosis and medical records for all aspects of breast cancer treatment including type of surgery. Kappa ranged from .89 (axillary dissection) to 1.0 (breast surgery). These self-report study issues are common to all kinds of survey research, whether Internet-based or not.

### Comparison With Prior Work

Our findings compare favorably with recent published data regarding rates of hysterectomy by modality. Wright et al [13] reported data from 2007-2010 in the national Premier Perspective Database and showed hysterectomy rates of 46.6% abdominal, 20.7% vaginal, 28.6% laparoscopic, and 4.1% robotic-assisted laparoscopic [13]. Our rates over the same time period (2007-2010) are 48.65% (958/1969) abdominal, 11.27% (222/1969) vaginal, 27.58% (543/1969) laparoscopic, 11.02% (217/1969) robotic, and 1.47% (29/1969) single-incision laparoscopic hysterectomy. Use of minimally invasive surgery was similar in the former and latter studies (53.43% [1052/1968] vs 51.35% [1011/1969], respectively).

Although prior studies of overall patient satisfaction after hysterectomy are scarce, and none include robotic surgeries, they have often reported greatest satisfaction with total laparoscopic minimally invasive surgery. McKenzie and Grant [14] prospectively compared total abdominal hysterectomy (TAH), laparoscopic-assisted vaginal (LAVH), and total laparoscopic hysterectomy (TLH) modalities on satisfaction scores (0-100) 3 months after the procedures and found no differences across these approaches. However, TLH patients indicated they were most satisfied (scores between 90 and 100: 79.6% TAH, 77.3% LAVH, 82.8% TLH). A study from Singapore showed that overall satisfaction (scale 1-10) was significantly higher for TLH compared with TAH patients (8.5 vs 7.2, *P*<.01) [15]. In a prospective study, Abdelmonem et al [16] found no differences in satisfaction rates one to three months after surgery by TAH, TLH, and total vaginal hysterectomy (TVH) approaches, but a greater percentage reported being highly satisfied in the TLH group compared with others (highly satisfied - physical: TAH 58%, TVH 67%, TLH 72%; highly satisfied - psychologically: TAH 63%, TVH 75%, TLH 81%) [16].

More recently, Sarlos and colleagues [17] reported findings from a randomized study indicating that patients who underwent robotic hysterectomy had significantly greater improvement in quality of life (*P*<.001) 6-weeks postoperatively than patients who had a conventional laparoscopic procedure. Investigators could not offer a good explanation for these results since most parameters such as incidence of complications, use of analgesics postoperatively, hospital length of stay, and return to activity and work were similar between the two surgical groups.

In a study following a group of premenopausal women for 8 years, some of whom eventually had a hysterectomy for benign pathology, Kuppermann et al [18] showed that multivariable predictors of greater satisfaction with hysterectomy included greater pelvic problem impact overall, higher scores before surgery on “benefits of not having a uterus,” and greater symptom reduction afterward. Although our study was not able to examine such factors related to satisfaction, these authors did not report the impact of surgical modality or return to normal activities on the outcome.

McKenzie and Grant [14] found that time to return to work was faster for patients who had LAVH and TVH than for TAH (no pain on movement took 3.4, 3.2, and 4.8 weeks, respectively; return to full activity without resting took 4.8, 5.5, and 6.6 weeks, respectively). Mean time to return to full activity was significantly shorter in TLH (mean 6.2 SD 6.3 weeks) versus TAH patients (mean 10.7 SD 6.3 weeks; *P*=.001). Abdelmonem et al [16] showed that recovery milestones (full mobility and return to work) were met significantly sooner after TVH and TLH compared with TAH. Return to work was shortest for TLH and TVH compared with TAH (mean 21.1 SD 10, mean 28.5 SD 13.3, mean 53.6 SD 11.8 days, respectively). A randomized study by Sarlos and colleagues [17] showed no differences between robotic and conventional laparoscopic hysterectomy in time until return to work or other activity.

In a prospective study, Vonk Noordegraaf and colleagues [19] found that the strongest influence on the amount of sick leave taken before returning to work after different types of gynecological procedures was the invasiveness of the surgery. Those who took the most leave had undergone an abdominal hysterectomy. Other predictive factors included expectations before surgery on return to work and preoperative functional assessment. Other factors that can have an effect on both return to work and satisfaction are receipt of clear and reasonable instructions and counseling on returning to normal activities [19,20]. Although our study did not ascertain this information, multivariable logistic analyses showed that return to work within 4 weeks was significantly less likely among women who had an abdominal (OR 0.25; 95% CI 0.22-0.28; *P*<.001) or a vaginal (OR 0.63; 95% CI 0.52-0.77; *P*<.001) hysterectomy. Being white (OR 1.61; 95% CI 1.36-1.91; *P*<.001) and earning a higher income (OR 1.12; 95% CI 1.06-1.19; *P*<.001) predicted an early return to work. Our analyses further show that return to walking within 2 days and return to driving within 1 week were also significantly less likely among women who had an abdominal hysterectomy.

The impact of patient experiences on others’ health care decisions has been explored in the literature, but studies are few. In an Internet study of factors involved in choosing between hospitals, investigators showed that the experience of other patients was considered at least as important in making a choice as information provided by the hospitals [21]. The patient-attributed “report card grade regarding physicians expertise” had the highest relative importance in making a choice. Overall, importance was highest for patient experience-based information on delivered care. The Pew research group has shown that other patients, family, and friends influence the choice of a treatment option, only second to the medical practitioner himself [22,23]. These US studies have found that when respondents were asked about who is more helpful when they need information about alternative treatment options, 63% indicated a medical professional, 24% indicated fellow patients, friends and family, and 4% indicated both equally. A more recent Pew study found that 70% of US adults got medical information, care, or support from a physician or health care professional the last time they had a health problem; 60% got information or support from friends and family; 59% looked on the Internet for health care information, and 24% got information or support from other patients [24].

Certainly more research is warranted to assess the impact of specific treatment modalities on patient experience and the impact of patient experience and satisfaction on others’ choices of medical and surgical treatments.

### Conclusions

Over the past decade, there have been several articles published on the topic of robotic surgery and its application to minimally invasive hysterectomy for the treatment of benign pathology. Those studies presented data from the perspective of noninferiority of robotic techniques compared with historically approved minimally invasive approaches and questioned the cost effectiveness of this technique. Other important considerations have been frequently omitted from the discourse in the literature. These factors include the impact of this technology on the quality of life of patients in terms of overall satisfaction with the surgical procedures and how quickly patients return to normal activities including being productive again in the workplace.

This retrospective study examined these parameters and compared patients’ responses by all the types of hysterectomy procedures currently being offered. It is acknowledged that in the population studied both laparoscopic and robotic hysterectomy rates increased while the rates of the abdominal and vaginal approaches decreased. From a clinical standpoint, any of the minimally invasive techniques would be preferable to an abdominal approach. It should be noted, however, that robotic hysterectomy was the only modality that was an independent predictor of better patient experience, greater satisfaction, and willingness to recommend and have the same procedure again.

## Abbreviations

IRB: institutional review board
LAVH: laparoscopic-assisted vaginal hysterectomy
OR: odds ratio
TAH: total abdominal hysterectomy
TLH: total laparoscopic hysterectomy
TVH: total vaginal hysterectomy
